# Adaptive Neuro-Fuzzy Inference System guided objective function parameter optimization for inverse treatment planning

**DOI:** 10.3389/frai.2025.1523390

**Published:** 2025-02-12

**Authors:** Eduardo Cisternas Jiménez, Fang-Fang Yin

**Affiliations:** ^1^Medical Physics Graduate Program, Duke University, Durham, NC, United States; ^2^Department of Radiation Oncology, Duke University Medical Center, Durham, NC, United States; ^3^Medical Physics Graduate Program, Duke Kunshan University, Kunshan, Jiangsu, China

**Keywords:** treatment planning system, fuzzy set theory, fuzzy inference system, Adaptive Neuro-Fuzzy Inference System, treatment plan parameters, artificial intelligence in radiotherapy planning, intensity-modulated radiation therapy

## Abstract

Intensity-Modulated Radiation Therapy requires the manual adjustment to numerous treatment plan parameters (TPPs) through a trial-and-error process to deliver precise radiation doses to the target while minimizing exposure to surrounding healthy tissues. The goal is to achieve a dose distribution that adheres to a prescribed plan tailored to each patient. Developing an automated approach to optimize patient-specific prescriptions is valuable in scenarios where trade-off selection is uncertain and varies among patients. This study presents a proof-of-concept artificial intelligence (AI) system based on an Adaptive Neuro-Fuzzy Inference System (ANFIS) to guide IMRT planning and achieve optimal, patient-specific prescriptions in aligned with a radiation oncologist's treatment objectives. We developed an in-house ANFIS-AI system utilizing Prescription Dose (PD) constraints to guide the optimization process toward achievable prescriptions. Mimicking human planning behavior, the AI system adjusts TPPs, represented as dose-volume constraints, to meet the prescribed dose goals. This process is informed by a Fuzzy Inference System (FIS) that incorporates prior knowledge from experienced planners, captured through “if-then” rules based on routine planning adjustments. The innovative aspect of our research lies in employing ANFIS's adaptive network to fine-tune the FIS components (membership functions and rule strengths), thereby enhancing the accuracy of the system. Once calibrated, the AI system modifies TPPs for each patient, progressing through acceptable prescription levels, from restrictive to clinically allowable. The system evaluates dosimetric parameters and compares dose distributions, dose-volume histograms, and dosimetric statistics between the conventional FIS and ANFIS. Results demonstrate that ANFIS consistently met dosimetric goals, outperforming FIS with a 0.7% improvement in mean dose conformity for the planning target volume (PTV) and a 28% reduction in mean dose exposure for organs at risk (OARs) in a C-Shape phantom. In a mock prostate phantom, ANFIS reduced the mean dose by 17.4% for the rectum and by 14.1% for the bladder. These findings highlight ANFIS's potential for efficient, accurate IMRT planning and its integration into clinical workflows.

## 1 Introduction

One of the most important stages in any intensity-modulated radiation therapy (IMRT) treatment planning process is inverse optimization (Oelfke and Bortfeld, 2001; Webb, 2019). Developing a high-quality treatment plan through inverse optimization requires finding optimal patient-specific Treatment Plan Parameters (TPPs). These include Weighting Factors (WFs), prescription doses (PDs), and dose-volume constraints. Effective plans are achieved when planners use treatment planning systems (TPS) to adjust and modify TPPs. This process is both repetitive and time-intensive (Hussein et al., 2018), relying on trial and error and user expertise (Hong et al., 2008). However, this process can be suboptimal due to time constraints, leaving potential room for improvement in the final plan. Therefore, an automated approach is highly desirable to assist in optimizing patient-specific TPPs for IMRT plans. Such approach can accommodate the variability in optimal trade-offs among patients and prevent the unintentional acceptance of suboptimal plans due to time constraints. Creating an IMRT plan involves a complex Multi-Criteria Optimization (MCO) process, as it requires balancing multiple TPPs (Lahanas et al., 2003; Monz et al., 2008). The MCO approach enables treatment planners and physicians to identify the optimal treatment plan for each patient by exploring and understanding the trade-offs. Although TPS can optimize the process using predefined TPPs, they cannot identify the optimal TPPs (Valdes et al., 2017; Feng et al., 2018). Consequently, identifying the optimal TPPs is a key challenge from the MCO perspective (Xing et al., 1999).

In a clinical context, achieving an optimal solution in treatment planning involves more than just minimizing an objective function based on predefined TPPs. It requires customization for each unique clinical case. This highlights the importance of incorporating “human expertise” in treatment planning, which can significantly reduce the time involved. Such expertise can be manifested through multiple PD levels, reflecting the physician's dosimetric intentions and offering flexibility in scenarios where achieving the ideal prescription level is unfeasible. While physicians strive to deliver 100% of the PD to the target and minimize doses to critical organs, this task is often challenging. Consequently, it may become necessary for physicians to collaborate closely with human planners to enhance and refine treatment plans based on initial results (Wang et al., 2019). Currently, human planners manually adjust TPPs and explore various PD levels for MCO. We propose a treatment planning method that employs a novel Artificial Intelligence (AI) system. This system can effectively and efficiently support planners and physicians in the planning process with minimal intervention, aiding in the identification of TPPs that meet the dosimetric goals while considering the varying prescription trade-off levels and objectives for each patient.

Significant progress has been made in recent years in automated, patient-specific treatment planning, with the aim of reducing manual intervention while enhancing the quality and consistency of treatment plans. Several TPS software companies have developed automation modules designed to replicate the decision-making processes of human planners during inverse optimization (Gintz et al., 2016). However, these approaches often rely on predefined (static) rules and templates, which may not always yield optimal outcomes for all patients. In response to these limitations, knowledge-based planning (KBP) has emerged as a promising approach. KBP leverages historical planning data in conjunction with patient-specific anatomical information to predict achievable plan quality for individual patients (Ge and Wu, 2019). The adoption of KBP for automated, individualized optimization in modulated radiotherapy has been demonstrated to improve plan quality while reducing variability among planners (Fogliata et al., 2017; Scaggion et al., 2018). A notable application of this approach is the integration of RapidPlan, a KBP-based tool, into the Eclipse™ TPS. RapidPlan utilizes dose-volume histogram (DVH) predictions derived from prior treatment plans to establish patient-specific optimization criteria. Its effectiveness has been validated across multiple studies (Fogliata et al., 2014; Hussein et al., 2016; Chang et al., 2016; Foy et al., 2017; Kubo et al., 2017), demonstrating its capacity to extract quantifiable knowledge from historical data and provide DVH guidance based on anatomical geometry analysis. This facilitates the development of optimized treatment plans, either through human planners or automated planning algorithms.

An alternative approach to patient-specific treatment planning is MCO (Craft et al., 2005, 2007), which simultaneously generates multiple “anchor” plans, each optimized for a distinct dosimetric objective. A Pareto surface is then created based on these anchor plans, representing the trade-offs between competing dosimetric objectives within a multidimensional space (Hoffmann et al., 2006; Serna et al., 2009). Physicians can continuously explore the possible treatment options, allowing them to identify the best possible plans by evaluating the dosimetric trade-offs represented on the Pareto surface, with the option to interpolate between anchor plans to refine the final selection. This MCO strategy has been successfully integrated into the RayStation TPS, providing valuable support to human planners in achieving preferred treatment outcomes. However, despite the advantages of automation programming interfaces, determining optimal TPPs remains essential in inverse planning.

Numerous investigations have explored approaches to determine optimal TPPs. One notable method involves using statistical analyses to identify relationships between TPPs and patient anatomy (Lee et al., 2013). Conversely, heuristic strategies leveraging voxel data from Computed Tomography have been proposed for TPP refinement (Wu et al., 2003; Yang and Xing, 2004; Yan and Yin, 2008; Wahl et al., 2016). Furthermore, genetic algorithms have also been employed to calculate WFs and ascertain the relative importance of multiple objectives (Wu and Zhu, 2001; Zhang et al., 2006). Remarkably, Deep Reinforcement Learning based on a virtual treatment planning framework has been developed, which has successfully identified TPPs and created plans comparable to those made by human planners (Shen et al., 2020). Despite these ongoing efforts to optimize TPPs, several questions remain unanswered. In particular, determining how to modify an existing plan to achieve desired dosimetric goals through TPP adjustments remains an open challenge. This requires the ability to predict plan changes resulting from varying TPP values within defined ranges, in order to accurately estimate the parameter modifications needed to satisfy dosimetric goals.

Our investigation presents a novel planning technique rooted in Fuzzy Inference Systems (FIS). Yan et al. incorporated fuzzy logic principles into the parameter optimization process during inverse planning (Yin et al., 2010). Within this technique, the design of the FIS is based on observations of how a human planner makes decisions during the planning process, relying on imprecise and non-numerical information. The decisions made by a human planner are translated into linguistic expressions, encapsulating their expertise in balancing trade-offs to find optimal TPPs. Initially, the authors employed FIS to determine an optimal prescription for the normal tissue in inverse treatment (Li and Yin, 2000). Subsequently, the researchers utilized a FIS to find the optimal TPPs for inverse planning, offering an alternative to traditional procedures overseen by human planners (Yan et al., 2003b,a). This FIS methodology was later incorporated into a clinical TPS (Yan et al., 2007). Upon evaluation of multiple clinical cases using this system, it was observed that the AI-driven dose plans based on FIS either matched or surpassed the quality of plans created by human planners usually.

While the outcomes from FIS applications were encouraging at the time, its clinical integration has been hindered by a lack of computational power and adaptability. The efficacy and precision of FIS rely on its core components: the membership functions (MFs) (i.e., fuzzy sets) and the rules (i.e., fuzzy rules). Unfortunately, these components are static and do not adjust well to new varying circumstances. Recent advancements in neural network (NN) research, coupled with significant improvements in computational capabilities, can overcome these existing obstacles. This breakthrough could elevate FIS to a central role in enhancing the optimization process for IMRT, supporting human planners in their decision-making processes.

The Adaptive Neuro-Fuzzy Inference System (ANFIS) has emerged as a promising method to address the limitations of FIS (Kar et al., 2014). ANFIS is a hybrid system that combines the principles of NN and FIS, offering a more dynamic and efficient methodology. At its core, ANFIS enhances FIS by incorporating a learning algorithm from NN theory. This enables ANFIS to fine-tune parameters, including fuzzy sets and fuzzy rules, using data samples. Compared to FIS, ANFIS demonstrates superior adaptability, flexibility, and efficacy in handling non-linear scenarios, resulting in more accurate outcomes.

Within the conventional IMRT planning framework, planners routinely assess plans generated by the optimization engine, iteratively fine-tuning TPPs to achieve an optimal plan. This study introduces a novel AI-driven methodology proof of concept, aiming to reduce the need for human intervention during the iterative IMRT planning phase. To realize this objective, we have merged an advanced FIS with ANFIS, culminating in the creation of an ANFIS Guided Inverse Planning (ANFIS-GIP) system. This system autonomously discerns necessary adjustments to TPPs, enhancing plan quality, thereby minimizing the dependence on manual input by human planners throughout the planning procedure.

## 2 Materials and methods

### 2.1 The ANFIS-GIP algorithm

ANFIS, is a type of artificial NN that is built upon the principles of FIS. This method was initially developed in the early 1990s (Jang, 1993). It enables a FIS to be represented as a NN, combining the structures and parameters of FIS with data-driven learning techniques algorithms found in NNs. The accuracy and computational complexity of the FIS model depend on the number and shape of the MFs, as well as on the number of rules and how they are evaluated. Initially, human experts construct a set of fuzzy IF-THEN rules, MFs and fuzzy logic operators based on their knowledge to emulate a precise problem-solving methodology. Subsequently, ANFIS refines the shapes of MFs and the evaluation of rules using sample data. The aim is to minimize the FIS's output error and boost accuracy. As a result, the FIS gains the ability to approximate nonlinear functions, providing it with a learning capability (Abraham, 2005).

**Figure 1:**
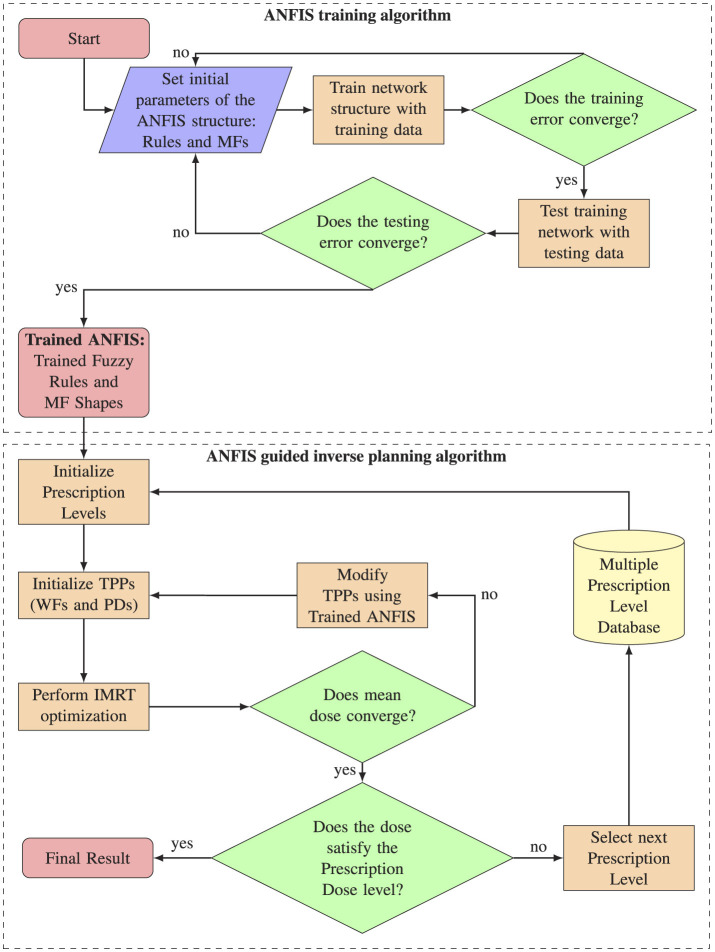
Complete workflow overview. The upper section illustrates the ANFIS training process algorithm, while the lower section details the algorithm for ANFIS-guided inverse planning.

We divided the AI algorithm into two sections, as illustrated in Figure 1. The first section comprises the ANFIS training algorithm, designed to effectively select the optimal parameters for FIS's MFs and how the rules are evaluated (rule strengths). The learning process is achieved by analyzing the correlation between input and output variables, as inferred from training datasets. After the system is trained and the optimal parameters for FIS are identified, the second section, named as ANFIS-GIP, concentrates on tailoring the dose distribution within the boundaries of clinical dosimetric goals. This process is achieved by adjusting the TPPs and probing patient-specific multiple prescription levels. The primary aim is to minimize the TPS objective function, which quantifies how effectively a treatment plan addresses its competing objectives.

#### 2.1.1 ANFIS training and architecture

For the ANFIS training algorithm, we generated the training dataset by recording the behavior of human planners during various treatment planning processes. Initially, the physician provides a set of PD levels for various structures. The human planner begins by modifying the TPPs based on these PD levels, starting with the most challenging level to achieve and then progressing to the easiest ones. These PD levels are then translated into intended dose points on a specific DVH. Subsequently, the TPS calculates a dose distribution (DD) based on the PD levels for various structures. The human planner further refines the prescriptions by adjusting the DVH dose points and WFs in the objective function for the planning target volume (PTV), organs at risk (OARs), and normal tissues (NTs). The TPS adapts in real time to these modifications, while the planner evaluates how well the new DVH aligns with their expected goals. The planner modifies the TPPs until the dosimetric goals are achieved. If the dosimetric goals are not met after modifying the TPPs, the planner repeats the process with the next PD level, continuously adjusting parameters until the desired dosimetric goals are achieved. With each modification, key data including the current DVH(t), PD(t), and WF(t) for each structure are saved. The DVH is recorded in intervals of 10% volume increments and stored in a database.

Following this, the input and output training sample datasets are calculated from the existing database. The input training data is defined as the difference between the prescribed dose, denoted as PD(t), and the computed dose, denoted as DVH(t). This difference represents how far the actual dose deviates from the physician's intended dose and is symbolized as ΔD(t).


(1) $$\begin{array}{l} \Delta \text{D}\left(t\right)=\frac{\text{DVH}\left(t\right)-\text{PD}\left(t\right)}{\text{PD}\left(t\right)} \end{array}$$

The training output is calculated as the relative difference between the PDs and the relative difference of the WFs for two subsequent adjustments, denoted by ΔPD(*t*) and ΔWF(*t*). These differences represent how the TPPs change over two consecutive steps.


(2) $$\begin{array}{l} \Delta \text{PD}\left(t\right)=\frac{\text{PD}\left(t+1\right)-\text{PD}\left(t\right)}{\text{PD}\left(t\right)} \end{array}$$

(3) $$\begin{array}{l} \Delta \text{WF}\left(t\right)=\frac{\text{WF}\left(t+1\right)-\text{WF}\left(t\right)}{\text{WF}\left(t\right)} \end{array}$$

The resulting dataset comprises nine primary vectors. Of these, three are assigned for input parameters (ΔD_PTV_, ΔD_OAR_, and ΔD_NT_), and six are allocated for output variables (ΔPD_PTV_, ΔPD_OAR_, ΔPD_NT_, ΔWF_PTV_, ΔWF_OAR_, and ΔWF_NT_). Based on these defined inputs and outputs, the optimal shapes of MFs and the evaluation of the fuzzy rules are determined using the ANFIS training algorithm. The goal is to generate a FIS with optimal parameters that can predict how much the TPPs need to change in order to minimize the difference between the actual dose and the dose intended by the physician.

After generating the training data, we developed an in-house ANFIS using PyTorch, a deep learning framework based on Torch, implemented in Python (Paszke et al., 2019). To compare the effectiveness of ANFIS with that of FIS, we similarly developed an FIS using Python's SciPy (Virtanen et al., 2020) and SciKit-Fuzzy libraries. The foundation for both the ANFIS and FIS, including the rules, initial MF shapes, fuzzy logic operators, and rule strengths, was adapted from US Patent 7804935B2 (Yin et al., 2010). We then took the FIS from the patent and represented it as an ANFIS, following the architecture proposed in the foundational ANFIS paper (Jang, 1993). The result of representing the FIS in the ANFIS architecture is as follows:

The proposed ANFIS architecture encompasses five distinct layers: (i) the Input MF layer, (ii) the Rule Layer, (iii) the Normalization Layer, (iv) the Defuzzification Layer, and (v) the Total Output Layer. It is important to note that within this architectural framework, only the first and fourth layers contain parameters that are trainable, which can be adapted using the provided input and output training data. The variables in Layer 1 are identified as premise parameters, whereas those in Layer 4 are designated as consequence parameters. Conversely, Layers 2, 3, and 5 are characterized by their non-trainable, fixed parameters. This training mechanism is aimed at reducing the discrepancy between the expected and the actual outputs during the training phase (Karaboga and Kaya, 2019).

i) *Fuzzy layer*: this layer is responsible for converting input values, specifically ΔD_PTV_, ΔD_OAR_, and ΔD_NT_, into fuzzy values. It accomplishes this by employing a MF that assigns these values to corresponding fuzzy sets: {PTV^high^, PTV^low^, OAR^high^, OAR^low^, NT^high^, NT^low^}, according to the eight specific rules *R*. The linguistic variables “*high*” and “*low*” are defined by corresponding MFs, denoted as $\mu_{{\text{PTV}}^{i}}$, $\mu_{{\text{OAR}}^{i}}$, $\mu_{{\text{NT}}^{i}}$. In this context, the linguistic variables represent the degree to which the calculated dose is *high* or *low* with respect to the intended PD level. Each node within this layer is adaptive and generates the output $O_{i}^{1}$


(4) $$O_{i}^{1}=\{\begin{array}{ll} \mu_{{\text{PTV}}^{i}}(\Delta {\text{D}}_{\text{PTV}}) & \text{ with }i=\text{low for }\{R_{1},R_{2},R_{3},R_{4}\}\\ \; & \text{ or }i=\text{high for }\{R_{5},R_{6},R_{7},R_{8}\}\\ \mu_{{\text{OAR}}^{i}}(\Delta {\text{D}}_{\text{OAR}}) & \text{ with }i=\text{low for }\{R_{1},R_{2},R_{5},R_{6}\}\\ \; & \text{ or }i=\text{high for }\{R_{3},R_{4},R_{7},R_{8}\}\\ \mu_{{\text{NT}}^{i}}(\Delta {\text{D}}_{\text{NT}}) & \text{ with }i=\text{low for }\{R_{1},R_{3},R_{7}\}\\ \; & \text{ or }i=\text{high for }\{R_{2},R_{4},R_{5},R_{6},R_{8}\} \end{array}$$

The output from each node reflects the membership degree within a specified linguistic category. The shape of the MF, which defines the linguistic label, is adjustable through node-specific parameters. These parameters are represented by the set {*a*_*i*_, *b*_*i*_}. A sigmoidal shape is adopted for the MFs. As an illustration, the functional representation for the node associated with PTVs is delineated as follows:


(5) $$\begin{array}{l} O_{i}^{1}=\mu_{{\text{PTV}}^{i}}\left(\Delta {\text{D}}_{\text{PTV}}\right)=\frac{1}{1+e^{-a_{i}\left(\Delta {\text{D}}_{\text{PTV}}-b_{i}\right)}}\text{for}i=R_{1},\ldots ,R_{8} \end{array}$$

ii) *Rule layer*: this layer consists of fixed nodes, each representing the firing strength, indicated as *w*_*i*_, associated with a specific fuzzy rule. Firing strength refers to the measurement of a rule's premise strength based on a given set of input values. It is calculated using fuzzy set operations to assess the activation level of the rule within a system. Nodes in this layer are responsible for calculating the firing strengths using the input values received from the preceding layer. The computation of firing strengths is carried out by


(6) $$\begin{array}{l} O_{i}^{2}=w_{i}=\mu_{{\text{PTV}}^{i}}\left(\Delta {\text{D}}_{\text{PTV}}\right)\times \mu_{{\text{NT}}^{i}}\left(\Delta {\text{D}}_{\text{NT}}\right)\times \mu_{{\text{OAR}}^{i}}\left(\Delta {\text{D}}_{\text{OAR}}\right)\\ \;fori=R_{1},\ldots ,R_{8} \end{array}$$

iii) *Normalization layer*: this layer consists of stationary nodes. Its primary function is to calculate the normalized firing strengths corresponding to each rule. This is achieved by determining the ratio of the firing strength of the *i*th rule to the sum of the firing strengths across all rules.


(7) $$\begin{array}{l} \bar{w_{i}}=\frac{w_{i}}{\overset{8}{\underset{i=1}{\sum }}w_{i}} \end{array}$$

iv) *Defuzzification Layer*: Each node within this layer is adaptive and receives two types of inputs: normalized firing strengths and the specific inputs ΔD_PTV_, ΔD_NT_, and ΔD_OAR_. The primary function of this layer is to produce weighted values for each rule's node, which are calculated by


(8) $$\begin{array}{l} \bar{w_{i}}f_{i}=\bar{w_{i}}\cdot \left(c_{i,0}+c_{i,1}\Delta {\text{D}}_{\text{PTV}}+c_{i,2}\Delta {\text{D}}_{\text{NT}}+c_{i,3}\Delta {\text{D}}_{\text{OAR}}\right)\\ \;\mathrm{for}\;i=R_{1},\ldots ,R_{8} \end{array}$$

where *c*_*i*_,, are the actual consequence parameters.

v) *Total output layer*: constituting a single, stationary node, this layer yields the ultimate output of the ANFIS. The process involves the summation of the outputs from each rule as obtained in the defuzzification layer.


(9) $$\begin{array}{l} f=\overset{8}{\underset{i=1}{\sum }}\bar{w_{i}}f_{i} \end{array}$$

**Figure 2:**
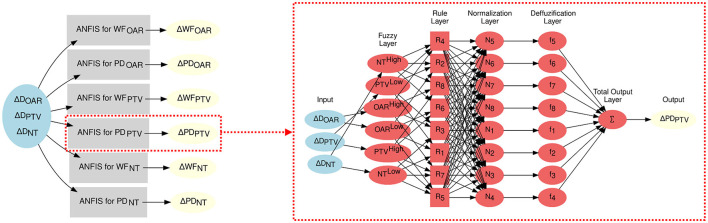
ANFIS-GIP network structure. **(Left)** Overall structure of the ANFIS-GIP. Each of the six TPPs possesses its own ANFIS network. **(Right)** Detailed structure of an ANFIS network that modifies the ΔPD_PTV_.

For each parameter to modify, the ANFIS needs to determine 12 premise parameters in the Fuzzy Layer, which dictate the shape of the input MFs. Additionally, it identifies four consequent parameters specific to the Defuzzification Layer. Figure 2 depicts the ANFIS NN used for determining the optimal parameters of the FIS. It is important to note that each of the six TPPs undergoing adjustments in the optimization process is regulated by an individual ANFIS network.

Due to the architecture of ANFIS, we chose to implement it using the PyTorch framework, which provides access to a wide array of optimizers, including the Recursive Least Squares (RLS) method, the Steepest Descent Method (SDM), and Back Propagation. These optimizers facilitate the optimization of both the premise and consequent parameters within the model. Additionally, the framework allows for the integration of other key features, such as experimentation with mini-batches, algorithms for optimizing learning rates, and a variety of loss functions. Since our output is a continuous variable, we use Mean Squared Error (MSE) as the loss function.

#### 2.1.2 ANFIS guided inverse planning

Upon successful training of the ANFIS, the next phase involves determining the optimal TPPs using the ANFIS-GIP algorithm. The workflow of the ANFIS-GIP algorithm is depicted in Figure 1. First, the PD level are set to the most challenging level defined by the physician, and the WFs for each structure are initialized to their default values. Following this, IMRT optimization is performed using the initial TPPs values. The IMRT optimizations were conducted with the VARIAN^®^ Eclipse™ TPS version 16.1.0, which integrates the Anisotropic Analytical Algorithm (AAA) 16.1.0. Tools such as DVH Estimation 16.1.0 and Photon Optimizer 16.1.0 were also used in this process. To facilitate communication between the ANFIS and FIS systems and the TPS, the Python Interface to Eclipse Scripting API (PyESAPI) was implemented, allowing dynamic interaction with the dose computation and optimization features of VARIAN^®^ Eclipse™.

The TPS optimization algorithm utilizes a quadratic cost objective function. This quadratic function comprises two primary TPPs that require fine-tuning to achieve the desired dose distribution (DD): the dose specifications (DS) and the WFs. The DS, typically set as dosimetric endpoints such as minimum and maximum dose values (*d*^min^, *d*^max^), represent the intended dosage for each structure. Meanwhile, the WFs act as penalties for either underdosing or overdosing the structures. The ideal DD is determined by minimizing the objective function, which is structured as follows:


(10) $$\begin{array}{l} \;F=\overset{\Omega_{\text{PTV}}}{\underset{i=1}{\sum }}{\text{WF}}_{\text{PTV}}^{\min}\cdot {\left[d_{\text{PTV}}^{\min}-d_{i}\right]}_{+}^{2}\\ +\underset{x\in \left\{\text{PTV},\text{OAR},\text{NT}\right\}}{\sum }\overset{\Omega_{x}}{\underset{i=1}{\sum }}{\text{WF}}_{x}^{\max}\cdot {\left[d_{i}-d_{x}^{\max}\right]}_{+}^{2} \end{array}$$

where *d*_*i*_ represents the calculated dose for each voxel *i*, while Ω_PTV_, Ω_OAR_, and Ω_NT_ denote the total number of voxels in the PTV, OAR, and NT, respectively. The term ${\text{WF}}_{\text{PTV}}^{\text{min}}$ denotes the penalty attributed to the underdosage of the PTV, and $d_{\text{PTV}}^{\text{min}}$ specifies the minimum dose (i.e., the lower objective) for the PTV. Finally,[·]_+_ is the positive operator which is defined as


(11) $${\left[x\right]}_{+}=xH(x)=\{\begin{array}{ll} x & x\geq 0\\ 0 & \text{ else } \end{array}$$

In this context, the lower objective involves applying the objective function to those doses that fall short of the desired dose value, thus defining the required dose levels in target structures. Additionally, ${\text{WF}}_{x}^{\max}$, represents the penalties associated with overdosing the structures. The parameter $d_{x}^{\max}$ designates the maximum permissible dose or the upper objective for these structures. The upper objective, $d_{x}^{\max}$, aims to cap the dose in any given structure, with the quadratic cost function being applied to doses exceeding the established dose value.

After performing the IMRT optimization, a convergence dose criterion is evaluated as follows:


(12) $$\begin{array}{l} \frac{\sqrt{{\left[\;{\text{D}}_{\text{PTV}}\left(n+1\right)-{\text{D}}_{\text{PTV}}\left(n\right)\right]}^{2}+{\left[{\text{D}}_{\text{OAR}}\left(n+1\right)-{\text{D}}_{\text{OAR}}\left(n\right)\right]}^{2}+{\left[{\text{D}}_{\text{NT}}\left(n+1\right)-{\text{D}}_{\text{NT}}\left(n\right)\right]}^{2}}}{\sqrt{{\text{D}}_{\text{PTV}}{\left(n\right)}^{2}+{\text{D}}_{\text{OAR}}{\left(n\right)}^{2}+{\text{D}}_{\text{NT}}{\left(n\right)}^{2}}}\\ \;<T \end{array}$$

Where *T* is a convergence constant, set at 0.01. This convergence criterion evaluates whether the change in mean doses between two consecutive TPP modifications reaches a plateau. ANFIS will continue to modify the TPPs until the convergence criterion is met. Once the convergence criterion is satisfied, the next step is to evaluate whether the dosimetric goals for the PD level are achieved. If the PD level is satisfied, the ANFIS-GIP algorithm terminates. Otherwise, the algorithm will take the next available PD level and restart the process of modifying the TPPs using ANFIS. The adjustment of TPPs in each iteration *i* is obtained as follows:


(13) $$\begin{array}{l} \;{\text{TPP}}_{i+1}={\text{TPP}}_{i}\cdot \left[1+\Delta \text{TPP}\right],\\ \text{TPP}\in \left\{{\text{WF}}_{\text{PTV}},{\text{WF}}_{\text{OAR}},{\text{WF}}_{\text{NT}},{\text{PD}}_{\text{PTV}},{\text{PD}}_{\text{OAR}},{\text{PD}}_{\text{NT}}\right\} \end{array}$$

In a clinical setting, achieving an optimal solution does not rely solely on minimizing the objective function modifying the TPPs; it also needs to be tailored to the specifics of each individual clinical case. This highlights why, in addition to identifying the optimal TPPs that minimize the objective function, incorporating “human expertise” can significantly reduce the time spent on treatment planning. Such human expertise can be conceptualized not only through the creation of the rules for the ANFIS and how these rules are evaluated, but also through the availability of patient-specific multiple dose prescription levels. These levels reflect varying physician intentions and provide flexibility in scenarios where achieving the desired prescription level proves unattainable. Table 1 illustrates how dose prescriptions can facilitate progress toward realistic AI-guided inverse planning optimization.

**Table 1:** Multiple dose prescriptions levels.

	**PTV**	**OAR**	**NT**
Level 1	100	20	30
Level 2	100	20	40
Level 3	100	30	40
Level 4	100	30	50
Level 5	100	40	50

The levels are represented as percentage of the Target dose prescription level.

### 2.2 Experiment design

We investigated the general learning patterns of ANFIS by conducting a simulation using non-clinical TG-119 C-Shape and mock prostate test phantoms (Ezzell et al., 2009; AAPM HQ Community Collection, 2023). To enhance our dataset and increase its diversity, we employed a routine to modified the phantom's CT structures using MONAI+ software (Consortium, 2024). Using the original phantoms' DICOM files, we introduced modifications to the positions and shapes of both the OAR and the PTV, resulting in a series of 150 new phantoms. By incorporating these modified phantoms, our objective was to enrich the dataset and have enough data for training and testing.

**Figure 3:**
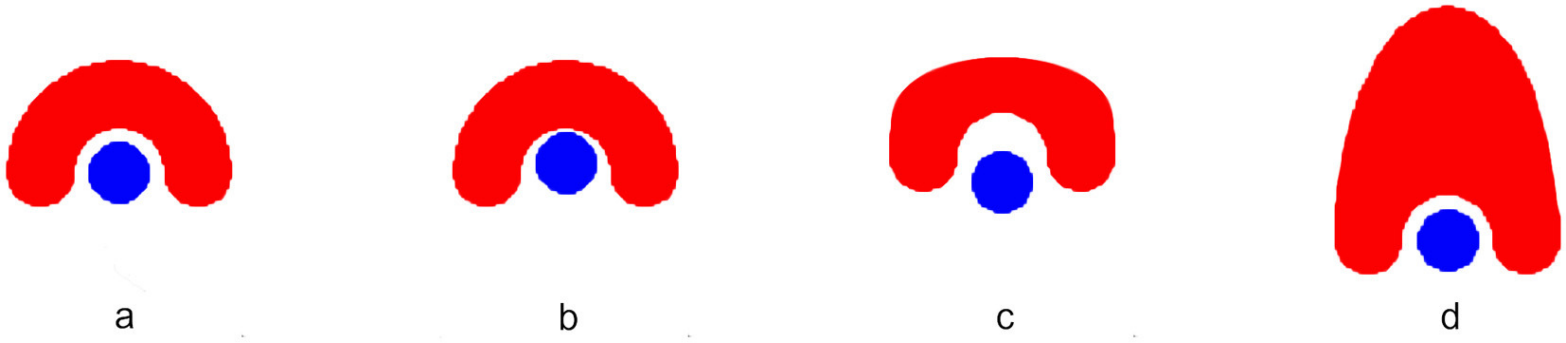
Examples of CT structure modifications in a phantom model. **(A)** The original C-shaped Target (red) symmetrically encloses the circular spinal cord (blue). **(B)** The spinal cord is shifted closer to the unchanged Target. **(C)** The Target is expanded in height and width, while the spinal cord is moved farther away. **(D)** The Target is stretched vertically, yielding a taller and narrower C-shape, with the spinal cord remaining in its original position.

Figure 3 shows an example of the CT structure modifications. Figure 3A shows the original phantom structures: the red structure represents the Target-a symmetrical, curved, dome-like shape enclosing the blue circular structure that represents the spinal cord. In Figure 3B, the red Target structure remains unchanged, and the spinal cord has been moved closer to the Target. In Figure 3C, the red Target structure has been expanded in height and width, and the spinal cord has been moved farther away. Finally, in Figure 3D, the red Target structure has been stretched vertically, creating a taller and narrower dome shape. This modification increases the height significantly compared to the original phantom while narrowing the lateral width. The spinal cord remains in the same position.

To establish the dataset, an experienced human planner meticulously crafted radiotherapy plans using modified phantoms, modifying the TPPs and proving the different PD levels. Then the input and output training data was calculated using the Equations 1, 2. Subsequently, the collected data samples were divided into two subsets: data generated by 60% of the phantoms formed the training set, with the remaining 40% allocated for validation. For the optimization process during model training, the Adaptive Moment Optimizer (Adam) algorithm was utilized, with an initial learning rate established at 0.01. This learning rate was subsequently adjusted downward in instances where no progress in reducing the training loss was observed. The Mean Squared Error (MSE) served as the loss function, augmented by an L2 regularization term (β = 0.015) applied to the weights of the model. The training regimen was initially set to process batches of 128 samples across a maximum of 500 epochs. However, due to the constrained size of the datasets used for calibration and cross-validation, the batch size was later modified to 16. To mitigate the risk of overfitting, an “EarlyStopping” criterion was implemented, halting training if no improvement in the loss was detected over a span of 20 epochs. The implementation of the models was carried out using Python version 3.8.13 and PyTorch version 1.10.1.

#### 2.2.1 Treatment plan evaluation

To evaluate a dosimetric comparison between the FIS and ANFIS-GIP systems, treatment plans were generated for 10% of the modified TG-119 C-Shape and mock prostate phantoms. Statistical comparisons of the results were performed using an unpaired two-sample *t*-test used where *p* < 0.05 indicates significance in the difference of the mean values. Plan quality was evaluated based on the original guidelines of AAPM Task Group 119 for the C-Shape phantom, while dosimetric goals based on the NRG Oncology RTOG 0126 protocol were followed for the mock prostate phantom, as follows:

*C-Shape Phantom*

- PTV_50.0 Gy_ ≥ 95%; PTV_55.0 Gy_ ≤ 10%
- OAR_25.0 Gy_ ≤ 5%

*Prostate Phantom*

- PTV_75.6 Gy_ ≥ 98%; PTVD_max_ ≤ 5%
- Rectum_75.0 Gy_ ≤ 15%; Rectum_70.0 Gy_ ≤ 25%; Rectum_65.0 Gy_ ≤ 25%; Rectum_60.0 Gy_ ≤ 50%
- Bladder_80.0 Gy_ ≤ 15%; Bladder_75.0 Gy_ ≤ 25%; Bladder_70.0 Gy_ ≤ 35%; Bladder_65.0 Gy_ ≤ 50%

To further compare the performance of ANFIS and FIS, we present the results for one plan generated using both systems. We used the original phantoms and obtained the final results for each PD level. The dose distribution, DVH, and dosimetric statistics are shown to illustrate these comparisons.

The FIS and ANFIS-GIP systems were evaluated using five distinct sets of dose prescriptions, delineated as [PTV, OAR, NT]: [100%, 20%, 10%], [100%, 25%, 10%], [100%, 30%, 10%], [100%, 35%, 10], and [100%, 35%, 15%] for the C-Shape, and [100%, 40%, 10%] for the Mock Prostate. These levels are represented as relative doses with respect to the PTV PD. The statistics evaluated include mean dose, standard deviation, and dose values covering 95% (D95) and 10% (D10) of all structures.

Percentage to Goal was also used to evaluate both systems. It is calculated as the percentage deviation of the achieved mean dose from the target dosimetric goal, expressed mathematically as:


(14) $$\begin{array}{c} \;\text{Percentage to Goal (\%)}\\ =\frac{\text{Achieved Mean Dose}-\text{Dosimetric Goal}}{\text{Dosimetric Goal}}\times 100 \end{array}$$

where the Achieved Mean Dose is the mean dose delivered to the structure (e.g., PTV, OAR, NT) during the treatment, and the Dosimetric Goal is the predefined target dose for that structure. Positive values (+%) indicate that the mean dose exceeds the goal, while negative values (-%) indicate that the mean dose is below the goal.

For the beam arrangement, the C-Shape phantom was evaluated using nine treatment beams, each delivering 6 MV photon beams. These beams were symmetrically distributed in a coplanar, 360-degree circumferential arrangement, positioned at 40-degree intervals from the vertical axis. This configuration is commonly used in spinal radiosurgery with IMRT. For the prostate mock phantom, a 6 MV, 7-field arrangement was applied, with beams spaced at 50-degree intervals from the vertical axis, following the RTOG 0126 protocol.

## 3 Results

**Figure 4:**
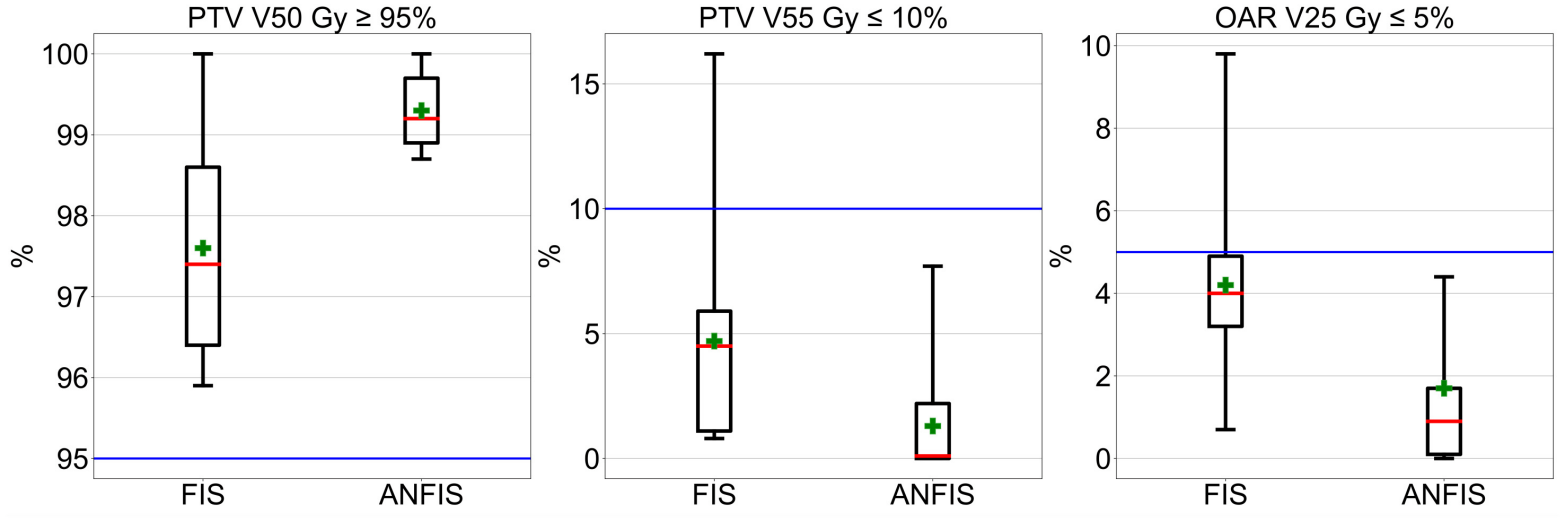
Box plot comparison of dosimetrical results for 10% of the augmented C-Shape phantoms.

Figure 4 and Table 2 present the dosimetric results comparing FIS and ANFIS plans for 15 modified C-Shape phantoms. The box plots in Figure 4 display a comparison between the two systems for the three dosimetric goals, while the numerical summaries of these metrics are provided in Table 2. We observe that the only dosimetric goal achieved by both systems in all 15 plans was the PTV 50Gy ≥ 95%. For this goal, FIS achieves a median coverage of 97.4%, with a mean of 97.6 ± 1.2% and an interquartile range (IQR) from ~96.6 to 98.6%, extending from 95 to close to 100%. ANFIS shows superior performance, achieving a tighter distribution with a median of 99.2%, a mean of 99.3 ± 0.3%, and an IQR between ~99.0% and 99.6%, suggesting greater consistency and reliability in reaching the target coverage compared to FIS. For PTV V55 Gy ≤ 10%, FIS has a median of 4.5%, a mean of 4.7 ± 3.8%, and shows high variability, extending up to 15%. ANFIS, however, achieves a much lower median of 0.1%, a mean of 1.3 ± 2.1%, and reduced variability, indicating greater effectiveness in meeting this constraint. Lastly, for OAR V25 Gy ≤ 5%, FIS yields a median of 4.0% and a mean of 4.2 ± 1.9%, with a broad range extending up to nearly 10%. In contrast, ANFIS demonstrates a lower median of 0.9%, a mean of 1.7 ± 1.7%, and significantly reduced variability, suggesting a better capability to minimize OAR exposure. The low *p*-values across all parameters indicate that the improvements observed with ANFIS over FIS are statistically significant on the difference of the mean doses, underscoring ANFIS's superior performance in meeting dosimetric goals.

**Table 2:** Numerical dosimetric results for 10% of the modified C-Shape phantoms, reported in the format: mean ± standard deviation (median).

**C-Shape phantom**				
**Structure**	**Parameters**	**FIS**	**ANFIS**	***p***
PTV	V 50Gy ≥ 95%	97.6 ± 1.2 (97.4)	99.3 ± 0.3 (99.2)	0.007
	V 55Gy ≤ 10%	4.7 ± 3.8 (4.5)	1.3 ± 2.1 (0.1)	0.000
OAR	V 25Gy ≤ 5%	4.2 ± 1.9 (4.0)	1.7 ± 1.7 (0.9)	0.001

Overall, these box plots and corresponding numerical values highlight that ANFIS outperforms FIS in meeting dosimetric goals for he C-Shape phantom, with greater consistency and reduced variability. ANFIS demonstrates a clear advantage in achieving target volume coverage while better adhering to dose constraints for both PTV and OAR, supporting its potential as a more robust approach for treatment planning.

**Figure 5:**
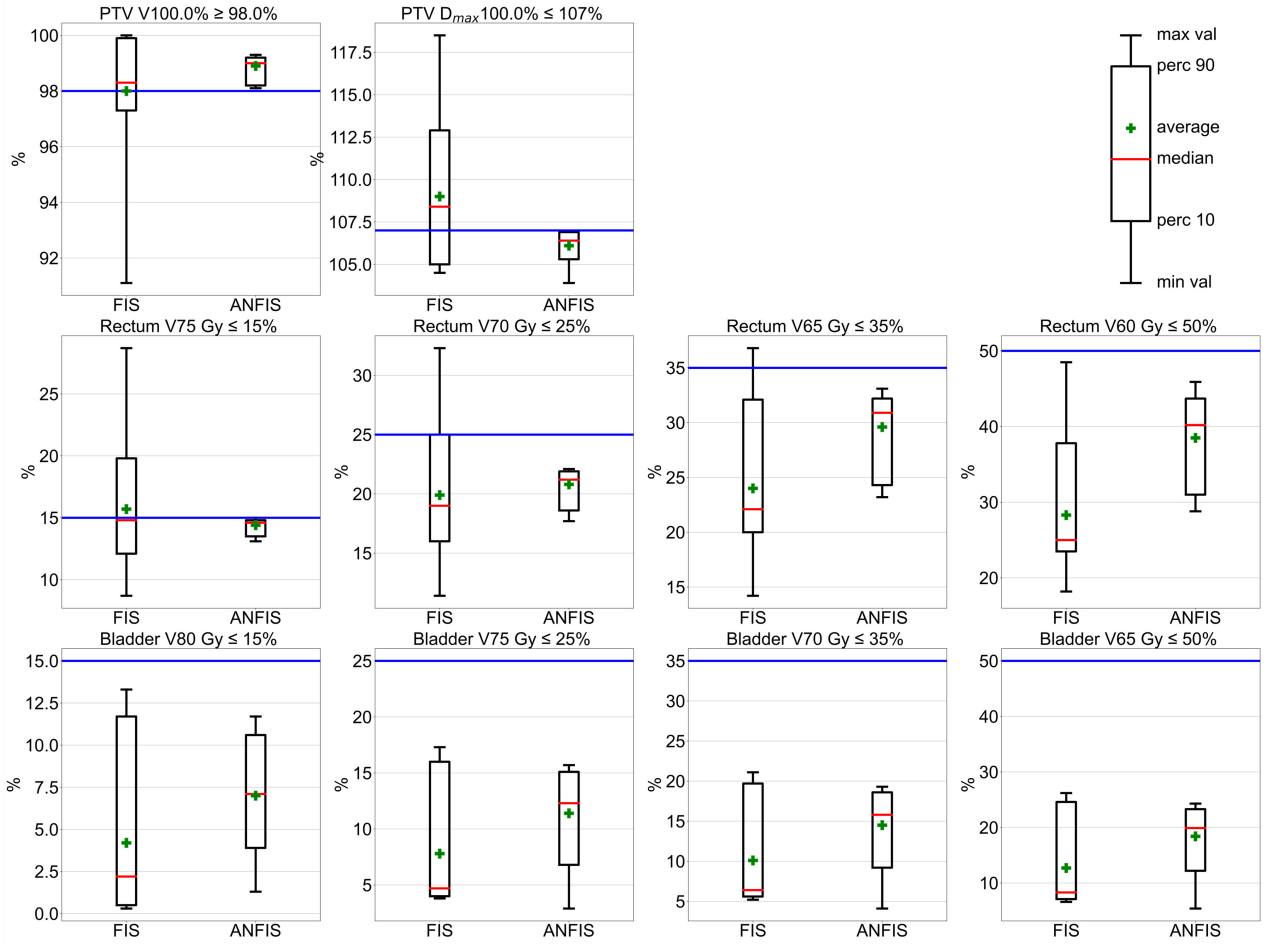
Box plot comparison of dosimetric results for mock prostate phantoms. The horizontal blue line represents the dosimetric goal.

Figure 5 presents a set of box plots comparing FIS and ANFIS plans across 10 dosimetric parameters for 15 modified mock prostate phantoms, with numerical summaries provided in Table 3. Each box plot illustrates the distribution of the percentage volume for the PTV and OAR constraints, focusing on compliance with clinical dosimetric goals.

**Table 3:** Numerical dosimetric results for 10% of the modified mock Prostate phantoms are reported in the format: mean ± standard deviation (median).

**Mock prostate phantom**				
**Structure**	**Parameters**	**FIS**	**ANFIS**	***p***
PTV	V 100% ≥ 98.0%	98.0 ± 2.2 (98.3)	98.9 ± 0.4 (99.0)	0.165
	D_max_ ≤ 107.0%	109.0 ± 3.7 (108.4)	106.1 ± 0.8 (106.4)	0.010
Rectum	V 75Gy ≤ 15%	15.7 ± 4.6 (14.8)	14.4 ± 0.6 (14.6)	0.293
	V 70Gy ≤ 25%	19.9 ± 4.9 (19.0)	20.8 ± 1.4 (21.2)	0.523
	V 65Gy ≤ 35%	24.0 ± 5.8 (22.1)	29.6 ± 3.2 (30.9)	0.004
	V 60Gy ≤ 50%	28.3 ± 7.8 (25.0)	38.5 ± 5.4 (40.2)	0.000
Bladder	V 80Gy ≤ 15%	4.2 ± 4.7 (2.2)	7.0 ± 3.0 (7.1)	0.077
	V 75Gy ≤ 25%	7.8 ± 5.3 (4.7)	11.4 ± 3.6 (12.3)	0.045
	V 70Gy ≤ 35%	10.1 ± 6.3 (6.4)	14.5 ± 4.2 (15.8)	0.038
	V 65Gy ≤ 50%	12.7 ± 7.85 (8.3)	18.4 ± 5.1 (19.9)	0.030

For the PTV goals, ANFIS is able to fulfill all of them, while FIS meets them in only some plans. For the PTV V100.0% ≥ 98.0% goal, ANFIS demonstrates a narrower distribution with a mean of 98.9 ± 0.4% and a median of 99.0%, while FIS achieves a mean of 98.0 ± 2.2% and a median of 98.3%. Only ANFIS was able to meet this goal across all plans, demonstrating greater consistency. For the PTV D_max_ ≤ 107.0% clinical goal, ANFIS outperforms FIS in maintaining this constraint, achieving a mean of 106.1 ± 0.8% and a median of 106.4%, whereas FIS has a mean of 109.0 ± 3.7% and a median of 108.4%, neither of which fulfill the goal, indicating greater variability and more frequent exceedances with FIS.

For the rectum goals, ANFIS is able to fulfill all four goals, whereas FIS, despite showing close results, only meets the easiest of these goals. For the rectum V75 Gy ≤ 15% goal, ANFIS demonstrates superior control with a mean of 14.4 ± 0.6% and a median of 14.6%, while FIS has a mean of 15.7 ± 4.6% and a median of 14.8%, displaying more variability and occasional constraint violations. For the V70 Gy ≤ 25% goal, ANFIS maintains this limit with a mean of 20.8 ± 1.4% and a median of 21.2%, while FIS has a mean of 19.9 ± 4.9% and a median of 19.0%, not fulfilling this goal across all 15 plans and showing higher variability in FIS results. For V65 Gy ≤ 35%, ANFIS consistently remains below this limit with a mean of 29.6 ± 3.2% and a median of 30.9%, while FIS has a mean of 20.0 ± 5.8% and a median of 22.1%, exhibiting increased variation. For the V60 Gy ≤ 50% goal, ANFIS achieves better compliance with the 50% volume constraint, with a mean of 38.5 ± 5.4% and a median of 40.2%, whereas FIS has a mean of 28.4 ± 7.8% and a median of 25.0%, indicating a larger spread in FIS values.

For the bladder goals, all four were achieved by both ANFIS and FIS. Nevertheless, ANFIS shows less variability, indicating a narrower range than FIS results. In general, the *p*-values indicate that ANFIS achieves statistically significant improvements on the differences of the mean dose value over FIS in several dosimetric constraints, particularly in controlling the maximum PTV dose and specific dose limits for the rectum and bladder. Overall, the dosimetric results indicate that ANFIS generally outperforms FIS in adhering to clinical dosimetric constraints, with lower variability and tighter control over both PTV and OAR metrics.

**Figure 6:**
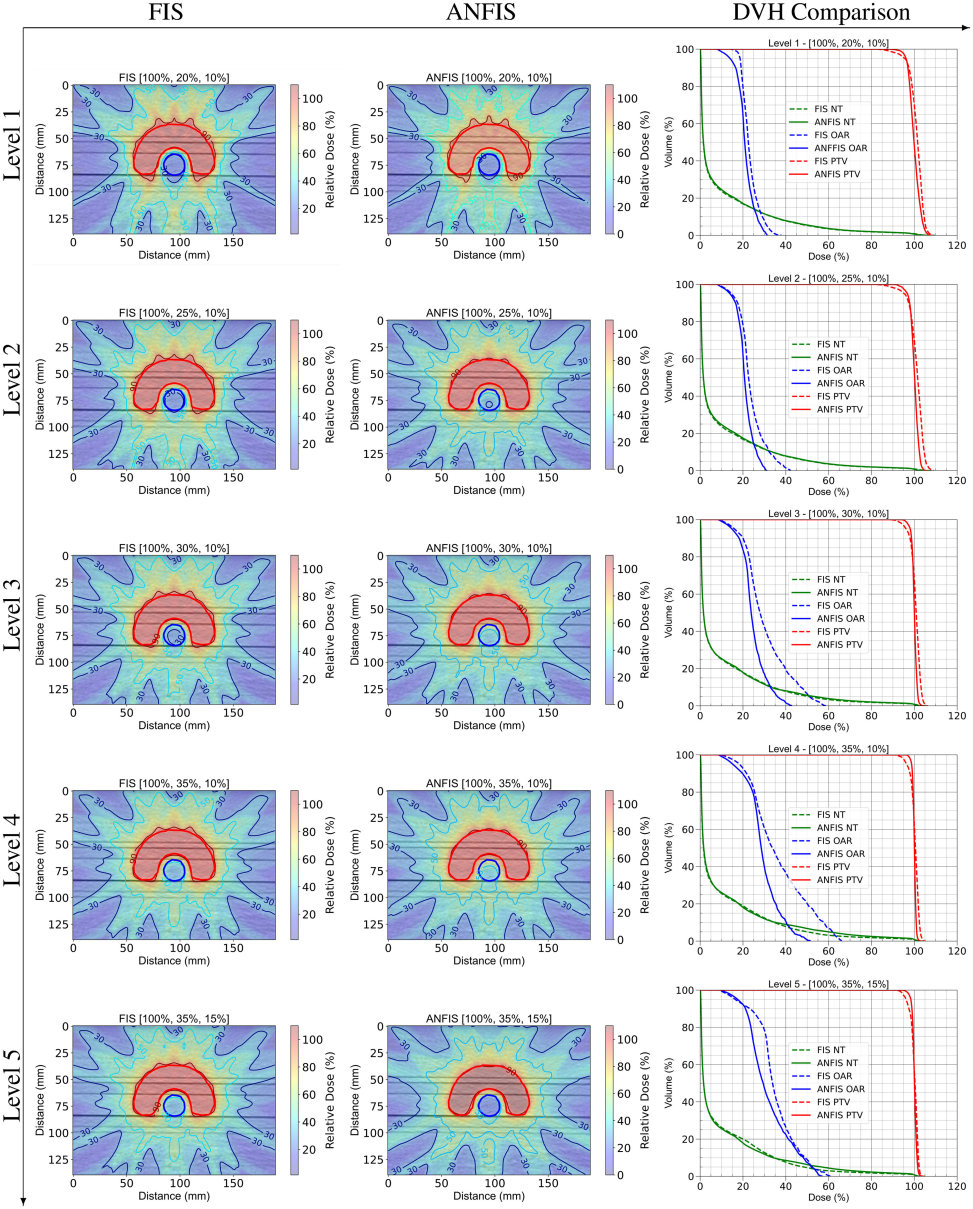
DD comparison between FIS and ANFIS for the C-Shape phantom, and DVH comparison across five dose prescription levels. Each row represents a specific PD level. In the DVH plots, dashed lines represent FIS, and solid lines represent ANFIS.

The next step in evaluating the performance of ANFIS vs. FIS was to compare an IMRT plan performed on the original phantoms, focusing on dose distribution (DD) and dosimetric statistics derived from their DVHs. Figure 6 presents a detailed comparison between FIS and ANFIS dose distributions for the C-Shape phantom across five PD levels, along with the corresponding DVH. Each row in the figure represents one dose prescription level, labeled from Level 1 to Level 5, and displays the best result achieved by each system at that particular level. The first two columns show the dose distributions for FIS (left) and ANFIS (center), with contour lines indicating relative dose levels as percentages of the PTV PD. The PD levels are specified at the top of each dose distribution plot in the format [100%, *X*%, *Y*%]. Additionally, the third column in each row presents the DVH comparisons for FIS and ANFIS, facilitating a comparison of how effectively each system meets the dosimetric goals for PTV coverage and OAR sparing across the different prescription levels.

Figure 6 shows differences in dose distribution patterns and dose coverage achieved by FIS and ANFIS, highlighting the potential of ANFIS to improve conformity to prescribed dosimetric goals across different dose levels. The dose distributions (DD) for Level 1 are very similar between the two systems, as observed in their respective DVHs. However, the DVHs suggest an improvement in OAR dose sparing with ANFIS. For Levels 2 through 5, there is a noticeable improvement in DD conformity to the PTV with ANFIS. The corresponding DVHs further suggest enhanced OAR dose sparing, especially at Levels 3 and 4.

Table 4 provides a comparison of dosimetric statistics for FIS and ANFIS across five different dose prescription levels for the C-Shape phantom. The best result achieved by each system at each prescription level is presented.

**Table 4:** Dose statistics comparing FIS and ANFIS for C-Shape phantom.

**Level**	**FIS—calculated dose (%)**								
	**PTV**				**OAR**			**NT**	
	**Mean**	**STD**	**D95**	**D10**	**Mean**	**STD**	**D10**	**Mean**	**STD**
1 [100, 20, 10]	101.1	3.3	95.5	104.6	23.3	3.9	29.0	10.0	18.9
2 [100, 25, 10]	101.1	3.4	95.3	104.7	35.3	12.4	26.4	10.0	18.9
3 [100, 30, 10]	100.6	2.3	96.2	103.1	40.6	13.7	32.8	10.2	18.8
4 [100, 35, 10]	100.3	2.0	96.3	102.4	36.7	13.4	57.8	10.4	18.7
5 [100, 35, 15]	100.2	1.8	96.6	102.1	32.2	8.7	44.4	10.5	18.7
**Level**	**ANFIS—calculated dose (%)**								
	**PTV**				**OAR**			**NT**	
	**Mean**	**STD**	**D95**	**D10**	**Mean**	**STD**	**D10**	**Mean**	**STD**
1 [100, 20, 10]	100.0	2.6	96.1	103.4	21.1	4.1	26.6	9.2	18.5
2 [100, 25, 10]	100.0	1.6	96.8	101.7	20.9	4.0	53.1	10.6	18.7
3 [100, 30, 10]	100.0	1.4	97.2	101.5	24.7	6.0	59.9	10.7	18.7
4 [100, 35, 10]	100.0	0.8	98.7	100.9	29.3	7.9	40.2	10.0	20.4
5 [100, 35, 15]	100.0	1.0	98.4	101.3	31.8	10.3	48.2	10.0	19.9

The table shows the best results obtained at each PD level.

For the PTV, ANFIS demonstrates slightly tighter control over dose delivery across all levels, with mean doses closer to the prescription (100%) and generally lower standard deviations compared to FIS. For example, at Level 5 [100, 35, 15], ANFIS achieves a mean of 100.0% with a standard deviation of 1.0%, while FIS shows a mean of 100.2% with a standard deviation of 1.8%. Additionally, ANFIS generally achieves higher D95 values, indicating more consistent target coverage. Notably, the median dose for the PTV aligns with the prescribed dose (PD) at all levels.

For the OAR, the results highlight ANFIS's superior sparing capabilities, as evidenced by lower mean doses and standard deviations across most levels. For instance, at Level 3 [100%, 30%, 10%], ANFIS achieves a mean dose of 24.7% with a standard deviation of 6.0%, compared to FIS's mean of 40.6% and a standard deviation of 13.7%. ANFIS also shows lower D10 values, reflecting improved control over high-dose regions in the OAR.

For the NT, both FIS and ANFIS show relatively consistent dose control, though ANFIS exhibits slightly lower standard deviations at certain levels. For example, at Level 4 [100, 35, 10], ANFIS achieves a mean NT dose of 10.0% with a standard deviation of 20.4%, while FIS shows a mean of 10.4% and a standard deviation of 18.7%.

Overall, Table 4 suggests that ANFIS generally outperforms FIS in achieving target dose conformity for the PTV, minimizing dose exposure to the OAR, and maintaining stable dose control for NT across different prescription levels. These results indicate that ANFIS offers improved consistency and control in adhering to dosimetric constraints. Notably, the ANFIS-GIP was able to fulfill the PD requirements for all structures at Level 4, meaning that the mean dose for each structure was less than or equal to the specified PD level. In contrast, FIS was unable to fulfill the PD requirements at any level.

**Figure 7:**
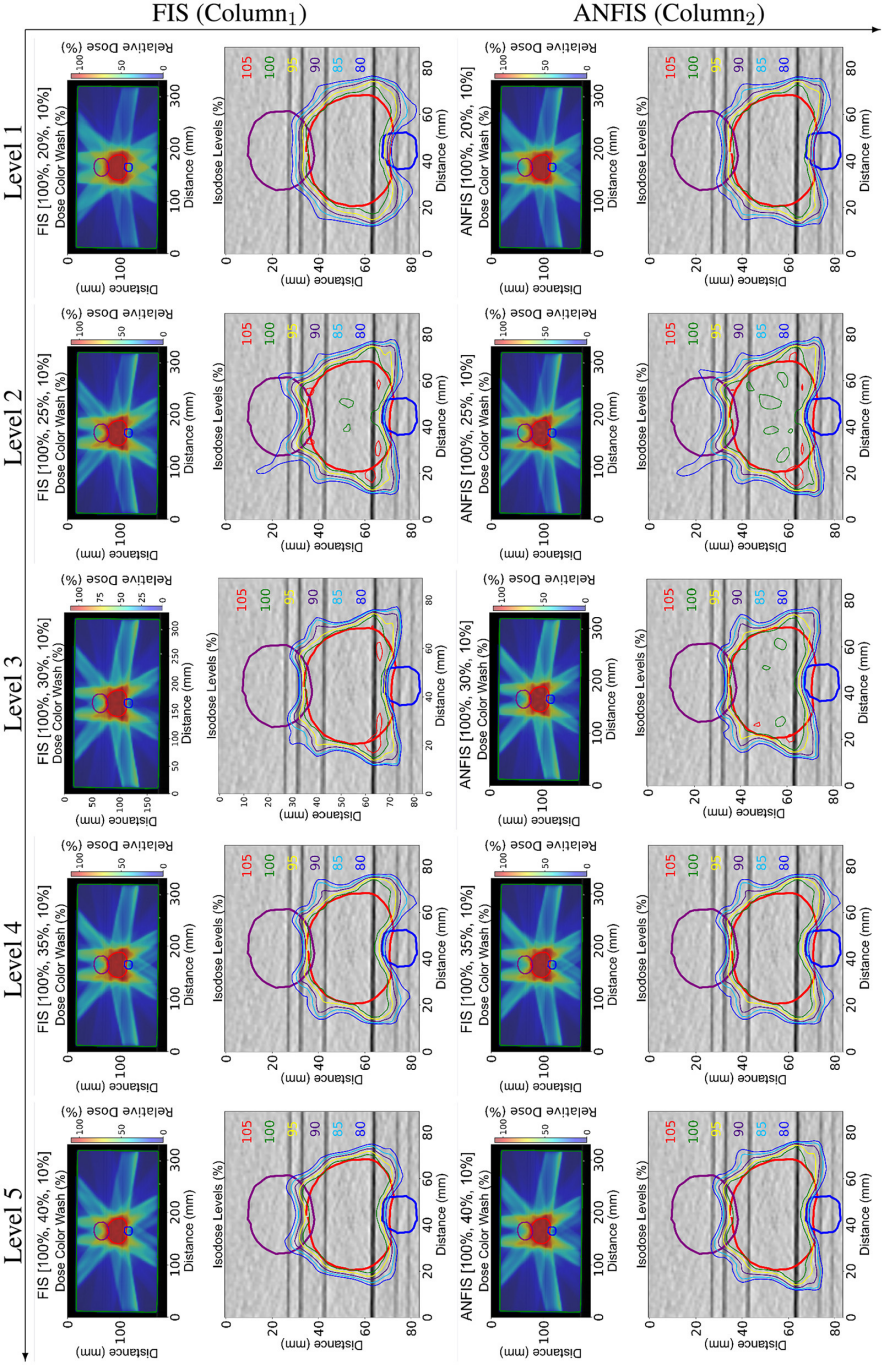
Dose distribution (DD) comparison between FIS (Column_1_) and ANFIS (Column_2_) for the mock prostate phantom across five dose prescription levels. Each row corresponds to a specific PD. The two columns show (1) the total dose distribution for the central slice and (2) a zoomed-in view of the same slice, illustrating the isodose curves around the relevant structures.

**Figure 8:**
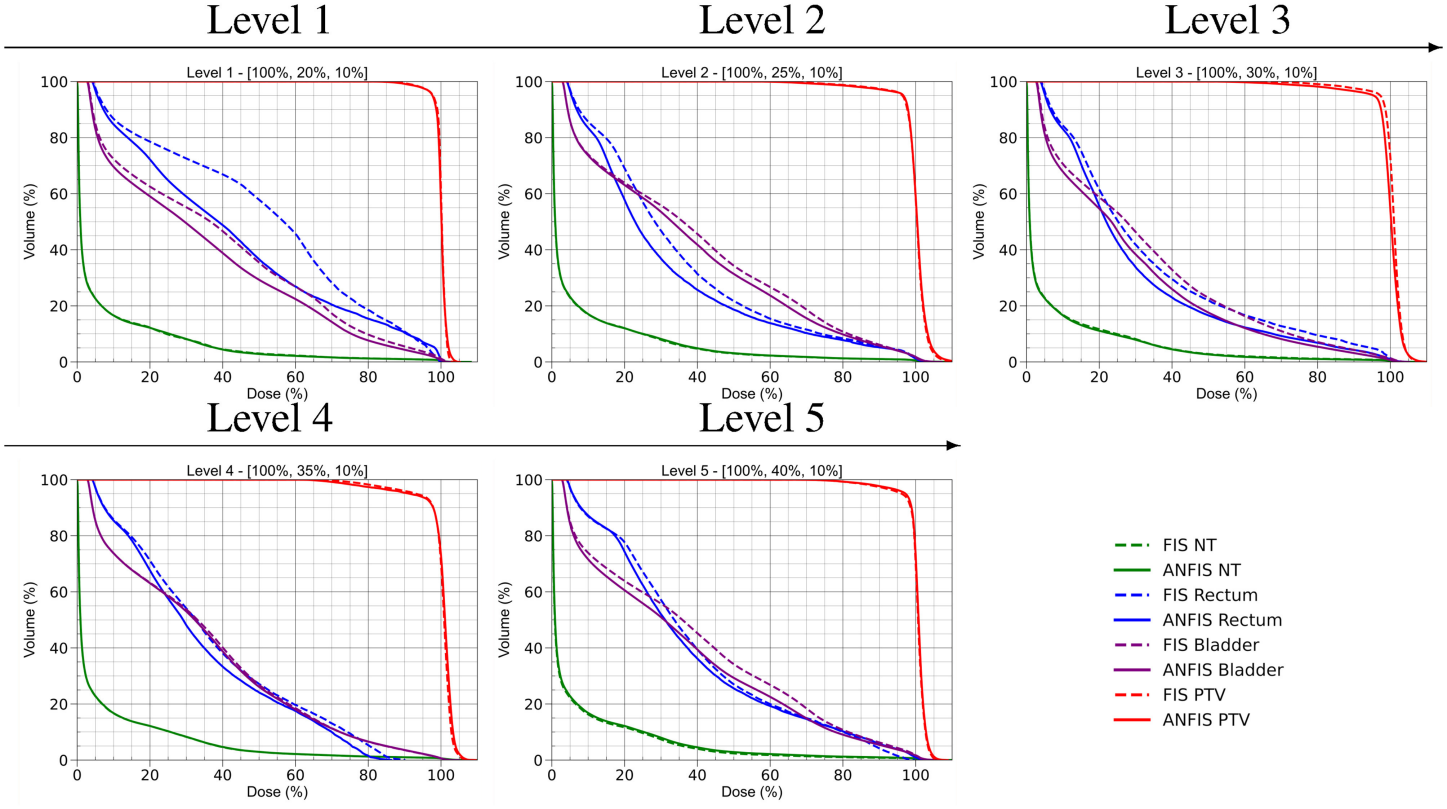
DVH comparison of FIS and ANFIS across the five prescription dose levels for the mock prostate phantom.

Figure 7 presents the complete dose distribution (DD) for the Prostate phantom along with a zoomed-in view of the isodose lines obtained with FIS and ANFIS for each PD level. Meanwhile, Figure 8 shows the corresponding DVHs, and Table 5 provides the dose statistics.

**Table 5:** Dose statistics comparing FIS and ANFIS for mock prostate phantom.

**Level**	**FIS—calculated dose (%)**													
	**PTV**					**Rectum (OAR)**			**Bladder (OAR)**			**Body (NT)**		
	**Mean**	**STD**	**D95**	**D10**	**D5**	**Mean**	**D30**	**D10**	**Mean**	**D30**	**D10**	**Mean**	**D30**	**D10**
1 [100, 20, 10]	100.0	1.7	97.8	101.5	101.8	51.8	68.8	90.9	38.3	55.8	79.6	7.0	2.4	24.5
2 [100, 25, 10]	100.0	4.2	96.3	103.1	104.1	35.0	41.4	74.0	39.1	56.0	82.0	7.1	2.4	24.8
3 [100, 30, 10]	100.0	4.5	94.4	102.4	102.9	34.7	42.1	73.1	38.4	53.6	79.9	7.0	2.4	25.4
4 [100, 35, 10]	100.0	4.6	93.0	102.8	103.5	36.6	46.8	74.2	34.8	47.2	72.3	7.1	2.4	25.6
5 [100, 40, 10]	100.0	3.5	96.1	102.7	103.4	38.8	47.1	80.1	38.7	55.4	81.5	6.6	2.2	24.1
**Level**	**ANFIS—calculated dose (%)**													
	**PTV**					**Rectum (OAR)**			**Bladder (OAR)**			**Body (NT)**		
	**Mean**	**STD**	**D95**	**D10**	**D5**	**Mean**	**D30**	**D10**	**Mean**	**D30**	**D10**	**Mean**	**D30**	**D10**
1 [100, 20, 10]	100.0	1.8	97.6	101.6	102.1	42.8	56.5	90.1	34.5	49.1	75.4	6.9	2.3	25.4
2 [100, 25, 10]	100.0	4.6	96.6	103.3	104.5	31.2	35.6	71.0	36.9	51.7	79.4	7.2	2.5	25.1
3 [100, 30, 10]	100.0	5.2	95.4	102.9	103.9	29.7	32.8	67.7	28.3	36.4	64.9	6.7	2.3	23.5
4 [100, 35, 10]	100.0	5.5	91.3	103.3	104.0	33.9	43.2	70.0	34.4	46.4	72.2	7.1	2.4	25.6
5 [100, 40, 10]	100.0	3.4	97.2	102.9	103.6	37.8	45.2	80.5	35.5	49.1	77.9	7.0	2.4	25.4

The table shows the best results obtained at each PD level.

The DD plots illustrate that ANFIS generally achieves a very similar DD within the PTV. The DVH for the PTV suggests that both FIS and ANFIS achieve comparable PTV coverage, with ANFIS demonstrating slightly tighter control in dose conformity, as evidenced by the steeper curves for the PTV. Based on the dose statistics, ANFIS maintains a mean dose close to 100% across all levels, with generally lower standard deviations compared to FIS. The D95 values for ANFIS are consistently higher than those for FIS, indicating improved target coverage. For FIS, the mean dose for the PTV is consistently 100% across all levels, with standard deviations ranging from 1.7 to 4.6. However, D95 values decrease from 97.8% at Level 1 to 93.0% at Level 4, indicating some variability in maintaining dose coverage at higher levels. In contrast, ANFIS maintains a mean dose of 100% for the PTV across all levels, with slightly better consistency in D95 values, ranging from 97.6 to 91.3%. While the standard deviation remains comparable to that of FIS, ANFIS shows tighter control over dose coverage.

Regarding OAR sparing (rectum and bladder), the DVHs indicate that ANFIS consistently provides better sparing for both organs, particularly at Levels 3 and 4, where ANFIS shows reduced dose exposure in the high-dose regions of the OARs compared to FIS. This suggests that ANFIS offers improved control in minimizing unnecessary dose to critical structures, as further illustrated in the dose distribution maps, especially in the lower part of the prostate and the upper region of the bladder.

For the rectum, the mean dose under FIS generally increases with prescription level, reaching a maximum of 38.8% at Level 5, with D30 and D10 values varying across levels, showing higher doses at lower prescription levels. In contrast, ANFIS consistently achieves lower mean doses to the rectum than FIS, especially at Levels 2 and 3, with mean doses dropping as low as 29.7%. Additionally, ANFIS shows reduced D30 and D10 values, particularly at Levels 3 and 4, indicating enhanced rectum sparing.

Similarly, for the bladder, FIS shows mean doses remaining around 38%–39% across levels, with D30 and D10 values fluctuating and reaching as high as 79.6% at Level 1. ANFIS, however, achieves lower mean doses to the bladder at each level, with a mean dose as low as 28.3% at Level 3. The D30 and D10 values for ANFIS are consistently lower than those for FIS, demonstrating improved bladder dose sparing.

For the NT both FIS and ANFIS produced similar results.

It is important to note that ANFIS is able to fulfill the PD levels from Level 3 through Level 5, meaning that the mean doses to the structures are lower than the prescribed PD level. In contrast, FIS was only able to achieve the PD requirement at the easiest level. In Table 5, for each prescription level, the mean dose for one OAR is consistently lower when using the ANFIS system compared to FIS, indicating that a reduced mean dose is achieved with ANFIS. However, for NT (i.e., the entire phantom body), the mean dose in Levels 2 and 3 is slightly lower with FIS. This result is not unexpected, given that the phantom body represents the largest structure, and its mean dose is averaged across the full volume of CT voxels. In contrast, smaller structures, such as organs, exhibit more pronounced differences in mean dose because of their fewer voxels. Similarly, in Table 5, the mean dose for both Rectum and Bladder (OARs) is lower with ANFIS for all prescription dose levels. Reducing the mean dose to OARs is clinically significant, as it decreases the probability of secondary cancer development.

**Figure 9:**
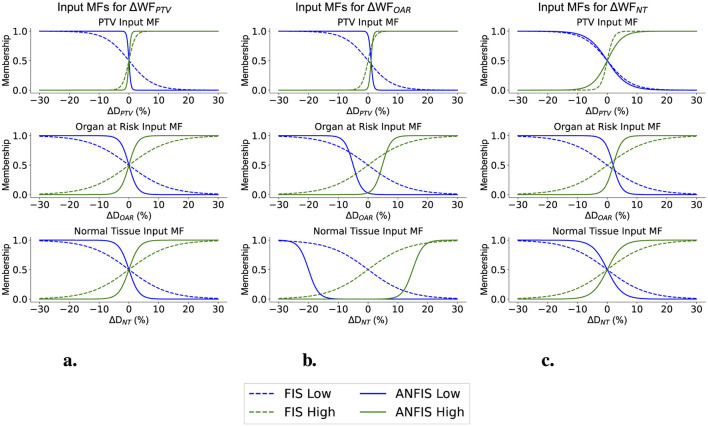
Input MFs for Modifying the WFs across different structures. **(A)** Input ΔWF_PTV_ MFs. **(B)** Input ΔWF_OAR_ MFs. **(C)** Input ΔWF_NT_ MFs.

**Figure 10:**
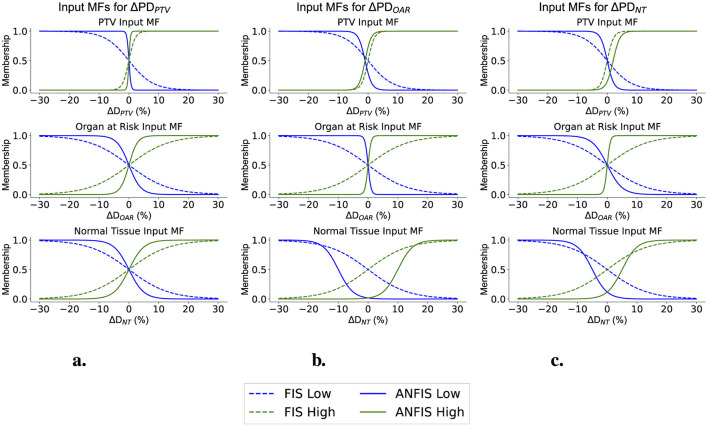
Input MFs for Modifying the PDs across different structures **(A)** Input MFs for ΔPD_PTV_. **(B)** Input MFs for ΔPD_OAR_. **(C)** Input MFs for ΔPD_NT_.

Finally, Figures 9, 10 illustrate how the membership functions (MFs) changed after ANFIS training. They display the input MFs used to modify the weighting factors (WFs) and prescription doses (PDs), respectively. The original FIS MFs are depicted with dashed lines, while the new optimal shapes identified after ANFIS training are represented by solid lines.

## 4 Discussions

Our initial findings indicate that the optimization of TPPs can be effectively achieved through the application of the ANFIS-GIP system. The dosimetric outcomes confirm that ANFIS enables a more accurate attainment of the desired DD compared to the FIS. The dosimetric comparisons show that ANFIS generally outperforms FIS, particularly in controlling the maximum dose to the PTV and displaying reduced variability. Additionally, the *p*-values reveal that ANFIS achieves statistically significant improvements in the mean doses. Despite ANFIS's overall superior performance across various levels, it was noted that at the most challenging level, the outcomes from ANFIS and traditional FIS were similar. However, at the remaining PD levels, ANFIS consistently demonstrated superior results. Notably, at any given prescription level, ANFIS was able to achieve a mean dose of 100% for the PTV. This outcome is due to the prioritization embedded in the rule set, which favors the PTV and OAR structures over the NT.

Tables 6, 7 present the percentage of goal attainment for each structure using FIS and ANFIS for the C-shape and mock prostate phantoms, calculated using Equation 14. A positive result indicates that the mean dose exceeds the dosimetric goal, whereas a negative result indicates that the mean dose is below the dosimetric goal. A prescribed dose (PD) level is reached when the percentage to goal is zero for the PTV and negative for the other structures.

**Table 6:** Percentages to goal obtained for the different structures for C-Shape pantom.

**Level (goals)**	**Percentage to goal (%)**					
	**PTV**		**OAR**		**NT**	
	**FIS**	**ANFIS**	**FIS**	**ANFIS**	**FIS**	**ANFIS**
1 [100, 20, 10]	+1.1	0.0	+16.5	+5.5	0.0	–8.0
2 [100, 25, 10]	+1.1	0.0	+41.2	–16.4	0.0	+6.0
3 [100, 30, 10]	+0.6	0.0	+35.3	–17.7	+2.0	+7.0
4 [100, 35, 10]	+0.3	0.0	+4.9	–16.3	+4.0	0.0
5 [100, 35, 15]	+0.2	0.0	–8.0	–9.1	–30.0	–33.3

**Table 7:** Percentages to goal obtained for the different structures for mock prostate phantom.

**Level (goals)**	**Percentage to goal (%)**							
	**PTV**		**Rectum (OAR)**		**Bladder (OAR)**		**Body (NT)**	
	**FIS**	**ANFIS**	**FIS**	**ANFIS**	**FIS**	**ANFIS**	**FIS**	**ANFIS**
1 [100, 20, 10]	0.0	0.0	+159.0	+114.0	+91.5	+72.5	–30.0	–31.0
2 [100, 25, 10]	0.0	0.0	+40.0	+24.8	+56.4	+47.6	–29.0	–28.0
3 [100, 30, 10]	0.0	0.0	+15.7	–1.0	+28.0	–5.7	–30.0	–33.0
4 [100, 35, 10]	0.0	0.0	+4.6	–3.1	–0.6	–1.71	–29.0	–29.0
5 [100, 40, 15]	0.0	0.0	–3.0	–5.5	–3.3	–11.3	–34.0	–30.0

Table 6 shows that for the PTV, ANFIS consistently achieved the target goal (0% deviation) across all levels, whereas FIS exhibited slight positive deviations at lower levels, such as +1.1% at Levels 1 and 2. For the OAR, ANFIS reduced the mean dose compared to FIS, with the most significant improvement observed at Level 3, where ANFIS achieved –17.7% compared to +35.3% for FIS. For NT, ANFIS achieved better dose reductions only at Levels 4 and 5, particularly at Level 5 (–33.3% for ANFIS compared to –30.0% for FIS). This outcome was expected because the mean dose is calculated over all CT voxels, meaning that ANFIS and FIS tend to yield similar results (which explains why the DVH for NT is nearly identical for each of the five levels). Overall, these results demonstrate that ANFIS effectively met dosimetric goals for the PTV while providing superior sparing of the OAR and NT. On average across the five levels, ANFIS outperformed FIS with a 0.7% improvement in mean dose conformity, while for the OAR, the mean dose was reduced by 28.8% when using ANFIS compared to FIS.

Table 7 presents the percentages to goal for the mock prostate phantom. For the PTV, both FIS and ANFIS consistently achieved the target goal (0% deviation) across all levels. For the rectum and bladder, ANFIS achieved lower mean doses than FIS at every level. For example, at Level 1, the rectum dose deviation was +114.0% for ANFIS compared to +159.0% for FIS, and for the bladder, the dose deviation was +72.5% for ANFIS vs. +91.5% for FIS. Additionally, for the body, ANFIS achieved slightly better reductions at Levels 1 and 3. On average across the five levels, ANFIS improved the percentage to goal for the mean dose by 17.4% for the rectum and by 14.1% for the bladder. Overall, Table 7 highlights ANFIS's superior ability to reduce mean doses for OARs and NT while maintaining accurate target coverage for the PTV.

We demonstrated that ANFIS-GIP can enhance the performance of an existing FIS. We believe that FIS has potential in a clinical setting, as it provides insight into the reasoning and decision-making process of the AI. It is important to note that in this proof of concept, the training of the ANFIS-GIP to optimize FIS parameters was based on PD levels, which explains why ANFIS-GIP primarily focuses on ensuring that the mean doses for the various structures are below the PD level targets. This approach can be further improved in the future by training the ANFIS-GIP system not only on PD levels but also on additional dosimetric goals. This enhancement could be achieved by incorporating new rules specifically targeting these additional parameters.

For the NT curves, only a minor difference is observed because the dose is averaged across the entire phantom, encompassing all CT voxels. Given that these phantoms are parallelepiped and do not replicate the anatomical variability of actual patients, the observed outcome aligns with expectations. Notably, for the OARs, the DVH curves for ANFIS remain below those for FIS, indicating a reduced dose, an outcome particularly desirable in radiation therapy.

Regarding the PTV volume, an optimal DVH curve aligns as closely as possible with the 100% dose line, indicating comprehensive dose coverage of the PTV. When comparing DVH data from Levels 3 to 5, the ANFIS-based plans demonstrate improved target coverage. Nevertheless, in Figure 8, the PTV DVH curves appear similar for both FIS and ANFIS, likely because the rules prioritize delivering the prescribed dose to the PTV before optimizing doses to other structures. This behavior is also evident in Table 5, where the mean PTV dose remains at 100% for all prescription levels. Despite these similarities for the target, the benefit of ANFIS is more pronounced in sparing OARs, which underscores its clinical advantage.

A critique of our proof of concept, based on the ANFIS-GIP algorithm workflow, is that the algorithm performs an IMRT optimization after each TPP modification, which can be time-intensive. However, the ANFIS-GIP system was designed to work in conjunction with the optimizer, allowing the TPS to respond in real-time to each TPP adjustment by ANFIS-GIP. Thus, the system is not intended to recalculate the final 3D dose volume after every TPP modification, thereby reducing computational demands.

Additionally, the ANFIS-GIP system was developed with the goal of reducing the time required for the planner to interact with the TPS. We envision this system as a starting point for human planners. In practice, ANFIS-GIP would first determine the optimal TPP modifications and achieve the best possible results for each PD level. Once the system has completed this process, the human planner could begin planning and refining the treatment from this optimized starting point.

The configuration of an ANFIS is crucial for successful application. In our system, the IF-THEN rules were derived from the expert knowledge of the treatment planner. For instance, the modification rules for the WFs can be described as follows: “if the PTV dose is below the PD, its WF should be increased; if the OAR and NT doses exceed the PD, their WF should also be increased.” These rules are broadly applicable for general cases without specific requirements. However, as additional clinical considerations are incorporated, the rule set may need to become more complex.

In clinical cases, various quantitative goals, such as maximum dose, dose coverage, and other dosimetric metrics, could be integrated into the ANFIS as inputs, replacing the calculated mean doses. Accordingly, the components of the fuzzy inference system (e.g., membership functions and rules) should be tailored to different clinical scenarios to ensure that the ANFIS responds appropriately to distinct input/output relationships. It should be noted that, in the current system, the rules were determined by a human planner. For practical purposes, it is also advisable to explore the automatic generation of rules based on the training data.

While adopting a strategy that replicates planner behavior might be considered a phenomenological approach, it provides a rapid pathway to establishing an automated planning process that can reliably achieve outcomes aligned with the goals set by human planners. Furthermore, our research illustrates that a FIS can evolve into an ANFIS through the analysis of training samples, reinforcing the adaptability and potential of such systems in the realm of treatment planning optimization.

Integrating intuitionistic fuzzy sets (Atanassov, 1986; Versaci et al., 2024) into the current fuzzy framework could substantially enhance the capabilities of our system by offering a more comprehensive means of modeling and managing the inherent uncertainty in treatment planning. Unlike traditional fuzzy sets, intuitionistic fuzzy sets incorporate not only the degree of membership but also the degree of non-membership and the level of indeterminacy. This added dimensionality could broaden the flexibility of the ANFIS approach, enabling a richer and more nuanced representation of complex decisions—particularly those involving intricate dose-volume constraints and the evaluation of optimal solutions. While implementing such a tool may not be immediately expected, considering this perspective for future developments could significantly improve the system's adaptability, resulting in more robust and accurate decision-making across various clinical scenarios.

In addition to exploring this integration, future work will focus on enhancing the adaptability and precision of the ANFIS-GIP system for broader clinical applications, specifically by applying it to real patient cases, such as prostate and head & neck treatments. Improvements will include expanding the rule set to incorporate additional dosimetric objectives, such as maximum dose constraints and dose homogeneity, for a more comprehensive optimization framework. Further training on diverse clinical datasets and testing in real-time treatment planning environments will be pursued to validate the system's robustness and efficiency. Additionally, we will compare ANFIS system performance against a human planner and other competing automated treatment planning AI methods, such as Rapidplan. Also, to measure the plan quality, we plan to use the VARIAN PlanScoreCard to automatically generate scoring metrics based on PTV coverage and awarding points for OAR doses below specified thresholds. Integrating a feedback loop for planners to fine-tune the system's output based on clinical experience could enhance its usability and acceptance in clinical workflows. A potential limitation of the proposed system lies in its reliance on training data drawn from human planner observations, which may introduce bias. Although the ANFIS system currently reflects the subjective expertise of a single planner, it could ultimately be enriched by integrating insights from multiple planners to reduce bias. For this proof of concept, modified data from a simple phantom case were used, but future work will focus on testing with real patient data, particularly for prostate cancer cases, and exploring the impact of automatically generating rules based on the dataset.

## 5 Conclusion

In this study, we present a novel proof of concept employing ANFIS for IMRT planning, which enables the generation of TPPs without human intervention. This approach facilitates an interactive process for treatment plan selection based on physicians' preferences and allows for the exploration of new Pareto frontier regions as needed. ANFIS demonstrated superior dosimetric outcomes compared to a traditional FIS, showing less variability and more robust results, as it consistently met all dosimetric goals. The methodology holds potential for enhancing compatibility with commercial TPS and automating IMRT optimization. By integrating human knowledge and “learning” from clinical data, this system reduces the need for manual input and emulates human planner decision-making, marking a significant advancement toward reducing the clinical workload.

## Data Availability

The raw data supporting the conclusions of this article will be made available by the authors, without undue reservation.
